# Mastering job interview skills for family physicians: Navigating the path to professional success

**DOI:** 10.4102/safp.v66i1.5852

**Published:** 2024-01-31

**Authors:** Chantelle C. van der Bijl, Arun Nair, Klaus B. von Pressentin

**Affiliations:** 1 Department of Family Medicine, Faculty of Health Sciences, University of the Free State, Bloemfontein, South Africa; 2 Department of Family Medicine, Robert Mangaliso Sobukwe Hospital, Kimberley, South Africa; 3 Department of Family, Community and Emergency Care, Faculty of Health Sciences, University of Cape Town, Cape Town, South Africa

*This Next5 contribution was informed by a conference workshop facilitated by the authors at the 25th Annual National Family Practitioners Congress in Johannesburg, South Africa.*

In the high-stakes realm of specialist family physician (FP) job interviews in South Africa, whether in the public or private sector, landing the ideal position boils down to more than just qualifications – it is about understanding the intricacies that make you the perfect candidate.

A good place to start this journey would be to identify potential employers of FPs, which include the district and sub-district health services (primary healthcare and intermediate care facilities, district hospitals and district clinical specialist teams), referral and specialised hospitals, universities, private sector and international opportunities. Keep your ear on the ground, scour platforms and tap into word-of-mouth and professional networks: websites of the provincial and national departments of health as well as the South African Academy of Family Physicians (SAAFP); vacancy circulars and block adverts; word of mouth from colleagues, mentors and heads of departments; WhatsApp groups and social media and organisational email groups such as SAAFP, World Organisation of Family Doctors (WONCA), the Primary Care and Family Medicine Network for Sub-Saharan Africa (PRIMAFAMED), private hospital and general practice networks and universities.

Your career decisions are deeply personal. Consider your current life roles (parent, guardian and life partner), interests and career aspirations. Think about how your choices align with family needs, urban vs. rural preferences and, importantly, your sense of belonging in the new work environment. Balance your professional ambitions with your life context and realities.

Once you have decided on which job opportunity to aim for, it is important to understand the description and requirements of the role you are interested in. Common themes include a sound knowledge of the district health and primary healthcare system, appropriate legislation, regulation and policies; good leadership and management skills; mentoring and conflict resolution skills, quality improvement and clinical governance; performance management of team members and cost containment measures.

Your *curriculum vitae* (CV) is your ticket to the interview (and not the job). The shortlisting phase of the recruitment process involves the recruitment and selection panel reviewing the CVs of applicants who meet the job requirements asked for in the advert. The CV is your marketing document, which should be concise yet informative and highlight the relevant experiences in a compelling narrative.

When (and not if) you are invited for the interview (as your well-crafted CV resonated with the employer’s needs), take time to prepare your verbal and non-verbal communication game plan. Non-verbal communication plays a pivotal, yet often underestimated, role. Beyond the common interview questions that probe qualifications and experience, the unspoken language of body gestures, facial expressions and tone of voice paints a nuanced portrait of the interviewee’s skills and confidence. Maintain confident eye contact to convey engagement and sincerity. Equally, a relaxed posture can denote comfort and approachability, vital qualities for building patient trust. Cultivate a relaxed posture, signalling comfort and approachability – qualities indispensable for building patient trust. Practice with simulated interviews; feedback refines your non-verbal cues, boosting your confidence significantly.^1^

**FIGURE 1:**
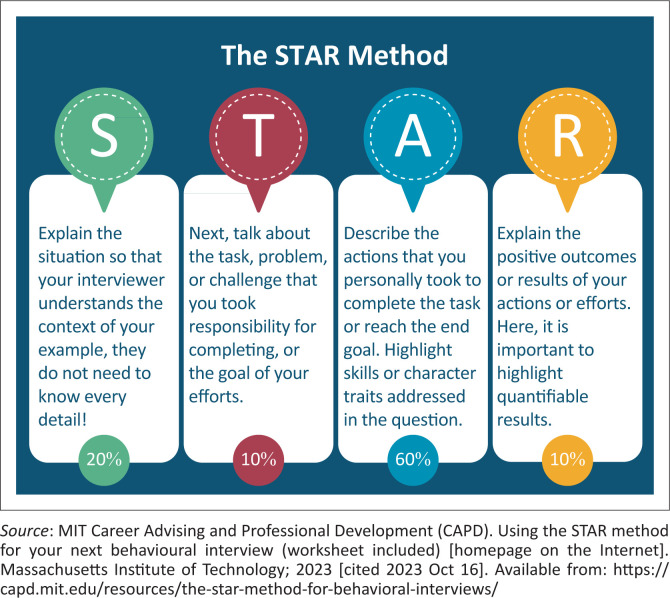
The Situation, Task, Action and Result method to answer behavioural interview questions.

Common interview questions tailored for FP roles revolve around clinical competency, decision-making under pressure and patient-centric attitudes. Common topics include questions on the health system, acute care, chronic care and clinical training or supervision. Preparing common interview questions and having an answer will prove to be helpful on the day.^2,3^ Utilise the *Situation, Task, Action and Result* (STAR) method to answer behavioural questions.^4^ Structure your responses to showcase your skills effectively by describing your approach to a previously experienced scenario by describing the components of the STAR acronym (see Figure 1).^4^ This method illuminates your approach by showcasing your interpersonal and leadership qualities.

In conclusion, securing a specialist FP post starts long before the day of the interview. Healthcare employers seek practitioners who embody both medical expertise and compassionate care. Importantly, having these qualities alone will not secure the position; it is your ability to communicate, both verbally and non-verbally, that seals the deal. It starts with understanding your own career aspirations and personal needs, aligning them with the job requirements and expressing them authentically.
